# Photon-counting CT for diagnosis of acute pulmonary embolism: potential for contrast medium and radiation dose reduction

**DOI:** 10.1007/s00330-023-09777-9

**Published:** 2023-06-14

**Authors:** Pauline Pannenbecker, Henner Huflage, Jan-Peter Grunz, Philipp Gruschwitz, Theresa S. Patzer, Andreas M. Weng, Julius F. Heidenreich, Thorsten A. Bley, Bernhard Petritsch

**Affiliations:** Department of Diagnostic and Interventional Radiology, University Hospital Würzburg, Oberdürrbacherstr. 6, D-97080 Würzburg, Germany

**Keywords:** Computed tomography angiography, Pulmonary embolism, Radiation exposure, Image enhancement, Contrast media

## Abstract

**Objective:**

To evaluate the image quality of an ultra-low contrast medium and radiation dose CT pulmonary angiography (CTPA) protocol for the diagnosis of acute pulmonary embolism using a clinical photon-counting detector (PCD) CT system and compare its performance to a dual-energy-(DE)-CTPA protocol on a conventional energy-integrating detector (EID) CT system.

**Methods:**

Sixty-four patients either underwent CTPA with the novel scan protocol on the PCD-CT scanner (32 patients, 25 mL, CTDI_vol_ 2.5 mGy·cm) or conventional DE-CTPA on a third-generation dual-source EID-CT (32 patients, 50 mL, CTDI_vol_ 5.1 mGy·cm). Pulmonary artery CT attenuation, signal-to-noise ratio, and contrast-to-noise-ratio were assessed as objective criteria of image quality, while subjective ratings of four radiologists were compared at 60 keV using virtual monoenergetic imaging and polychromatic standard reconstructions. Interrater reliability was determined by means of the intraclass correlation coefficient (ICC). Effective dose was compared between patient cohorts.

**Results:**

Subjective image quality was deemed superior by all four reviewers for 60-keV PCD scans (excellent or good ratings in 93.8% of PCD *vs.* 84.4% of 60 keV EID scans, ICC = 0.72). No examinations on either system were considered “non-diagnostic.” Objective image quality parameters were significantly higher in the EID group (mostly *p* < 0.001), both in the polychromatic reconstructions and at 60 keV. The ED (1.4 *vs*. 3.3 mSv) was significantly lower in the PCD cohort (*p* < 0.001).

**Conclusions:**

PCD-CTPA allows for considerable reduction of contrast medium and radiation dose in the diagnosis of acute pulmonary embolism, while maintaining good to excellent image quality compared to conventional EID-CTPA.

**Clinical relevance statement:**

Clinical PCD-CT allows for spectral assessment of pulmonary vasculature with high scan speed, which is beneficial in patients with suspected pulmonary embolism, frequently presenting with dyspnea. Simultaneously PCD-CT enables substantial reduction of contrast medium and radiation dose.

**Key Points:**

*• The clinical photon-counting detector CT scanner used in this study allows for high-pitch multi-energy acquisitions.*

*• Photon-counting computed tomography allows for considerable reduction of contrast medium and radiation dose in the diagnosis of acute pulmonary embolism.*

*• Subjective image quality was rated best for 60-keV photon-counting scans.*

**Supplementary Information:**

The online version contains supplementary material available at 10.1007/s00330-023-09777-9.

## Introduction

Acute pulmonary embolism (PE) is a frequent and potentially lethal condition. Timely diagnosis and therapy is crucial for optimizing the clinical outcome. In the first decade of the twenty-first century, computed tomography pulmonary angiography (CTPA) has become the reference standard in the diagnostic workup for suspected acute PE due to high sensitivity, ubiquitous availability, and short acquisition time [1–4]. In recent years, dual-energy (DE) CT has proven to be of incremental value in the diagnosis of PE by offering additional spectral information and reducing the required radiation dose [5, 6]. Even further functional information is gained via various post-processing applications that exploit the fact that elements with high atomic numbers like iodine (*n* = 53) possess different absorption characteristics at varying energy levels [7, 8]. In the imaging of PE, two post-processing applications are of particular significance: visualization of the pulmonary perfusion in color-coded iodine-distribution-maps [9] and virtual monoenergetic imaging (VMI), which means that images are reconstructed at a virtual monoenergetic level (keV). Especially monoenergetic low-keV images have been shown to improve image quality in low-contrast settings [10]. In the past, various technical approaches for DE-CT imaging have been developed, where as technical commonality all of them are based on an energy-integrating detector (EID) [11].

With the introduction of the first clinical photon-counting detector (PCD) CT, a novel multi-energy-CT approach has recently become available [12]. In directly converting PCD-CT, the specific energy level of every individual incoming photon is measured [13]. Among the most important advantages of the PCD technology are the suppression of electronic noise below predefined thresholds, higher spatial resolution with preserved dose efficiency, and full spectral information even in high-pitch scans. Notably, the impact of these theoretical advantages on iodine contrast and radiation dose requirements in patients with pulmonary embolism has not been evaluated to date.

Therefore, the aim of this study was to assess the image quality of a high-pitch PCD-CTPA scan protocol with ultra-low contrast medium and radiation dose for the diagnosis of acute pulmonary embolism and compare its performance to an established DE-CTPA protocol on a third-generation dual-source CT scanner.

## Material and methods

### Patient population and study design

The local institutional review board approved this study and waived the need for written informed individual consent. In this retrospective investigation, a total of 64 patients who underwent CTPA between October 2021 and March 2022 for diagnostic workup of suspected acute pulmonary embolism were included. Of those, 32 individuals received their examinations on a clinical PCD-CT scanner (Naeotom Alpha, Siemens Healthcare GmbH), whereas the other 32 CTPAs were performed on an EID-based dual-source DE-CT system (Somatom Force, Siemens Healthcare GmbH). For every patient, age, gender, height, and weight, as well as lateral and anterior–posterior chest diameter were documented. In addition, body mass indices and effective thoracic diameter were calculated. Detailed information regarding the study population are summarized in Table 1.

**Table 1:** Demographic data of patients with suspected pulmonary embolism

	PCD-CT	EID-CT	*p* value
Patients (*n*)	32	32	
Pulmonary embolism (*n*/%)	10/31.3%	7/21.9%	
Female/male (*n*)	20/12	18/14	
Age (± SD) [years]	65.2 (± 17.1)	71.0 (± 14.0)	n.s
BMI	27.6 (± 6.0)	26.8 (± 5.7)	n.s
Lateral chest diameter (± SD) [cm]	35.5 (± 5.1)	36.5 (± 7.3)	
a.p. chest diameter (± SD) [cm]	24.8 (± 5.1)	26.1 (± 3.3)	
Effective chest diameter (± SD) [cm]	29.6 (± 3.3)	30.8 (± 4.6)	n.s
Scan length (± SD) [mm]	304 (± 6.4)	329 (± 49.8)	n.s
Scan time (± SD) [s]	0.58 (± 0.02)	2.59 (± 0.4)	< 0.001

### CT scan protocols

PCD-CT scans were performed on a first-generation clinical PCD-CT scanner that employs dual-source beam geometry and is equipped with two cadmium-telluride photon-counting detectors (QuantaMax, Siemens Healthcare GmbH) offering a 50-cm field-of-view (FOV; primary PCD array). EID scans were performed on a third-generation 192-slice dual-source CT scanner equipped with latest EID technology (Stellar, Siemens Healthcare GmbH) offering a 35.3-cm FOV in dual-energy mode.

In the patient group examined on the PCD-CT system, acquisition parameters were determined to achieve approximately half the radiation dose of our well-established dual-energy CTPA acquisition protocol. Tube potential was 120 kV using the “QuantumPlus” scan mode in a high-pitch setting (pitch of 2.0) with an image quality setting of “BQ 50.” In the EID group, tube potential was set to 90 kV/Sn 150 kV with a reference tube current–time product of 60/46 mAs which represents our dual-energy acquisition protocol in daily routine. The helical pitch factor was set to 0.55 for DE image acquisition. Automated tube-current modulation (CARE Dose 4D, Siemens Healthcare GmbH) was applied for all examinations in both patient groups.

Before each scan, a predefined amount of iodinated contrast medium was applied (Imeron® 350, Bracco) via an antecubital vein using an automated injector. In the EID group, 50 mL of contrast medium was administered with a flow rate of 4 mL/s, corresponding to an iodine delivery rate of 1400 mg/s [14, 15]. Based on recent studies discussing a potential for contrast medium dose savings in PCD-CT by up to 50% while still obtaining diagnostic attenuation [13, 16], we aimed for a significant reduction in terms of halving the dose of our standard dual-energy acquisition protocol. In doing so, we approached the lower limit of 25 mL step by step, starting from our standard dose (supplementary figure). Due to shorter acquisition time and a smaller bolus of contrast medium, we adapted flow rate and trigger threshold of our injection protocol to ensure a sufficient bolus length. As a result in the PCD group, 25 mL was injected with an iodine delivery rate of 875 mg/s as an “ultra-low contrast medium approach.” Detailed parameters of the applied CT scan protocols are provided in Table 2.

**Table 2:** Acquisition and reconstruction parameters

	PCD-CT	EID-CT
Scan mode	Quantum Plus	Dual-source/dual-energy
Collimation	144 × 0.4	2 × 96 × 0.6 mm (z-flying focal spot)
Rotation time [s]	0.25	0.25
Pitch	2.0	0.55
Spectral information FOV [cm]	50.0	35.3
Tube potential [kV]	120	90/Sn150 on tube A/B
Tube current time product (ref.) [mAs]	BQ 50	60/46 mAs on tube A/B
Automatic tube current modulation	on	on
Automatic tube potential control	N/A	N/A
Amount of contrast medium [mL]	25	50
Flow rate [mL/s]	2.5	4.0
Trigger threshold [HU]	100	120
Iodine delivery rate [mg/s]	875	1400
Matrix size [mm × mm]	Auto-Matrix	512 × 512
Slice thickness [mm]	3.0	3.0
Kernel polychromatic images/T3D	Bv36	Br40
Kernel monoenergetic images at 60 keV	Bv36	Qr40

*PCD-CT* photon-counting detector CT, *EID-CT* energy-integrating detector CT, *FOV* field-of-view, *Sn* tin, *HU* Hounsfield units

For study purposes, two stacks of axial images (3-mm slice thickness) were reconstructed for each examination: (1) polychromatic low-energy threshold 120-kV images referred to as “T3D” by the vendor for PCD-CT or blended series mimicking a single-energy 120-kV scan for EID-CT and (2) monoenergetic image series at 60 keV for both detector technologies. All images were reconstructed using specific iterative image reconstruction algorithms (ADMIRE Br40/Qr40 for EID-CT; QIR Bv36 for PCD-CT) at a strength level of 3. Detailed reconstruction parameters are provided in Table 2.

### Evaluation of objective image quality

For analyzing and comparing the objective image quality of CTPAs, the mean CT attenuation in Hounsfield units was assessed for the pulmonary vasculature (pulmonary trunk, left upper lobe artery, right lower lobe artery), the descending aorta, and the periscapular musculature (left teres major muscle) by placing standardized circular regions of interest (ROIs) in 3-mm axial images at corresponding sites both in 60 keV and polychromatic 120 kV images. The periscapular musculature was chosen as reference tissue as it shows homogenous attenuation without relevant contrast enhancement in the arterial phase [17]. If a pulmonary embolism was present, the ROI was placed proximal to the embolus. The ROIs were drawn by one reader (PP, 2 years of clinical experience in CT imaging). In accordance to previous publications by other research groups, the contrast-to-noise ratio (CNR) and the signal-to-noise ratio (SNR) were calculated for each CTPA in the 60 keV and the polychromatic images using the following formulas [17, 18]:

$$SNR=\frac{ROI\;vessel\;\lbrack HU\rbrack}{Image\;noise\;vessel\;(SD\;of\;HU)}$$

$$CNR=\frac{ROI\;vessel\;\left[HU\right]-ROI\;muscle\;\lbrack HU\rbrack}{Image\;noise\;vessel\;(SD\;of\;HU)}$$

### Evaluation of subjective image quality

Four radiologists (R1-4) with different expertise in the field of cardiovascular CT imaging (reader 1, P.P., 2 years; reader 2, P.G., 5 years; reader 3, H.H., 7 years; reader 4, B.P., 12 years) assessed the subjective image quality of every 60 keV and polychromatic CTPA using a 4-point rating scale. Readers were blinded regarding both scanner type and energy level. Rating scores were determined as follows: (1) excellent overall image quality, no motion artifacts, excellent CTPA contrast, excellent diagnostic confidence down to peripheral branches, no relevant subjective image noise; (2) good overall image quality, minor motion artifacts, good CTPA contrast, good diagnostic confidence down to subsegmental level, minor motion artifacts, minor subjective image noise; (3) moderate to poor overall image quality, severe motion artifacts, just barely acceptable CTPA contrast, practicable evaluation to at least segmental level, high subjective image noise, still of diagnostic quality; (4) non-diagnostic image quality.

### Radiation exposure

Both the volume computed tomography dose index (CTDI_vol_) (mGy) and the dose-length product (DLP) (mGy·cm) were recorded from the dose report automatically generated by the CT scanner. For estimation of the effective radiation dose (ED), a conversion factor of 0.018 mSv/mGy·cm was applied. For PCD scans, size-specific dose estimates (SSDEs) were recorded as calculated by the scanner, whereas for EID scans, SSDEs were calculated as described previously [19].

### Statistical analysis

Dedicated statistical software (SPSS Statistics for windows, version 25, IBM) was used for all statistical analyses. *p* values < 0.05 were considered statistically significant. To compare continuous variables (presented as means ± standard deviations), the Mann–Whitney *U* test was used. Subjective image ratings are presented as absolute numbers and relative frequencies. For comparison of scale ratings, the Mann–Whitney *U* test was used as described previously [20]. Intraclass correlation coefficients (ICCs) were calculated in order to evaluate inter-reader reliability. Moreover, the percentage of CTPA scans (at T3D/120 kV and at 60 keV) rated with a score of 1 (= excellent image quality) or 2 (= good image quality) was calculated for each reader.

## Results

### Patient population

Of the 64 patients included, 38 were women (20 in the PCD group *vs.*18 in the EID group) and 26 were men (12 in the PCD group *vs.* 14 in the EID group). The mean age was 65.2 (± 17.1) years in the PCD group and 71.0 (± 14.0) years in the EID group. The mean BMI was 27.6 (± 6.0) kg/m^2^ in the PCD cohort *vs.* 26.1 (± 3.3) kg/m^2^ in the EID group. The incidence of PE was *n* = 10 (31.3% of cases) in the PCD group *vs*. *n* = 7 (21.9% of cases) in the EID group. Characteristics of the respective patient collectives are summarized in Table 1.

### Objective image quality

CT attenuation values within relevant vascular structures were substantially higher in the EID group than in the PCD group, both in the polychromatic images and in the 60-keV images: the pulmonary trunk (316.1 ± 111.3 [PCD, 60 keV] *vs.* 586.3 ± 162.1 [EID, 60 keV], *p* < 0.001; 239.4 ± 78.3 [PCD, T3D] *vs.* 430.2 ± 117.7 [EID, blended], *p* < 0.001), the right lower lobe artery (336.2 ± 120.0 [PCD, 60 keV] *vs.* 556.5 ± 154.9 [EID, 60 keV], *p* < 0.001; 256.4 ± 84.0 [PCD, T3D] *vs.* 424.9 ± 117.6 [EID, blended], *p* < 0.001), and the left upper lobe artery (331.1 ± 120.7 [PCD, 60 keV] *vs.* 534.1 ± 148.1 [EID, 60 keV], *p* < 0.001; 250.1 ± 87.4 [PCD, T3D] *vs.* 410.2 ± 25.7 [EID, blended], *p* < 0.001). Also, CT attenuation values for the descending aorta were significantly higher in the EID group than in the PCD group (179.4 ± 72.6 [PCD, 60 keV] *vs.* 304.2 ± 122.1 [EID, 60 keV], *p* < 0.001; 141.3 ± 52.1 [PCD, T3D] *vs.* 230.3 ± 86.4 [EID, blended], *p* < 0.001). There was no significant difference in CT attenuation values of periscapular musculature between PCD and EID scans with 57.9 ± 8.9 *vs.* 56.0 ± 6.5 for 60 keV images (*p* = 0.277) and 52.2 ± 6.5 *vs.* 54.4 ± 6.0 for T3D/blended images (*p* = 0.428).

Both SNR and CNR were significantly higher for EID scans in 60 keV, as well as in the blended image series for all evaluated vessel sections, e.g., in case of the pulmonary trunk 30.6 ± 8.6 (SNR)/27.7 ± 8.6 (CNR) in the 60 keV EID scans *vs*. 17.6 ± 4.8 (SNR)/14.2 ± 4.8 (CNR) in the 60 keV PCD scans (all *p* < 0.001). A detailed comparison of all ROI measurements and SNR/CNR calculations is provided in Table 3.

**Table 3:** Objective image quality

	PCD-CT	EID-CT		PCD-CT	EID-CT	
	T3D	Blended 120 kV	*p* value	60 keV	60 keV	*p* value
Pulmonary trunk						
CT attenuation (± SD) [HU]	239.4 (± 78.3)	430.2 (± 117.7)	< 0.001	316.1 (± 111.3)	586.3 (± 162.1)	< 0.001
SNR (± SD)	17.1 (± 4.4)	29.5 (± 8.1)	< 0.001	17.6 (± 4.8)	30.6 (± 8.6)	< 0.001
CNR (± SD)	13.2 (± 4.3)	25.7 (± 8.0	< 0.001	14.2 (± 4.8)	27.7 (± 8.6)	< 0.001
Right lower lobe artery						
CT attenuation (± SD) [HU]	256.4 (± 84.0)	424.9 (± 117.6)	< 0.001	336.2 (± 120.0)	556.5 (± 154.9)	< 0.001
SNR (± SD)	19.7 (± 8.7)	32.2 (± 10.2)	< 0.001	20.2 (± 7.5)	36.4 (± 13.6)	< 0.001
CNR (± SD)	15.6 (± 7.8)	27.2 (± 9.4)	< 0.001	16.5 (± 6.9)	30.2 (± 9.6)	< 0.001
Left upper lobe artery						
CT attenuation (± SD) [HU]	250.1 (± 87.4)	410.2 (± 110.9)	< 0.001	331.1 (± 120.7)	534.1 (± 148.1)	< 0.001
SNR (± SD)	19.3 (± 6.4)	25.7 (± 10.7)	0.006	20.7 (± 8.4)	28.8 (± 11.1)	0.002
CNR (± SD)	15.0 (± 5.8)	20.3 (± 9.1)	0.001	16.8 (± 7.8)	23.9 (± 10.1)	< 0.001
Descending aorta						
CT attenuation (± SD) [HU]	141.3 (± 52.1)	230.3 (± 86.4)	< 0.001	179.4 (± 72.6)	304.2 (± 122.1)	< 0.001
SNR (± SD)	11.6 (± 6.2)	18.0 (± 7.1)	< 0.001	11.4 (± 6.1)	18.1 (± 7.7)	< 0.001
CNR (± SD)	7.4 (± 5.3)	13.7 (± 7.0)	< 0.001	7.8 (± 5.4)	14.7 (± 7.8)	< 0.001
Muscle						
CT attenuation (± SD) [HU]	52.2 (± 6.5)	54.4 (± 6.0)	n.s	57.9 (± 8.9)	56.0 (± 6.5)	n.s

*PCD-CT* photon-counting detector CT, *EID-CT* energy-integrating detector CT, *SD* standard deviation, *SNR* signal-to-noise ratio, *CNR* contrast-to-noise ratio, *HU* Hounsfield units

### Subjective image quality

**Fig. 1:**
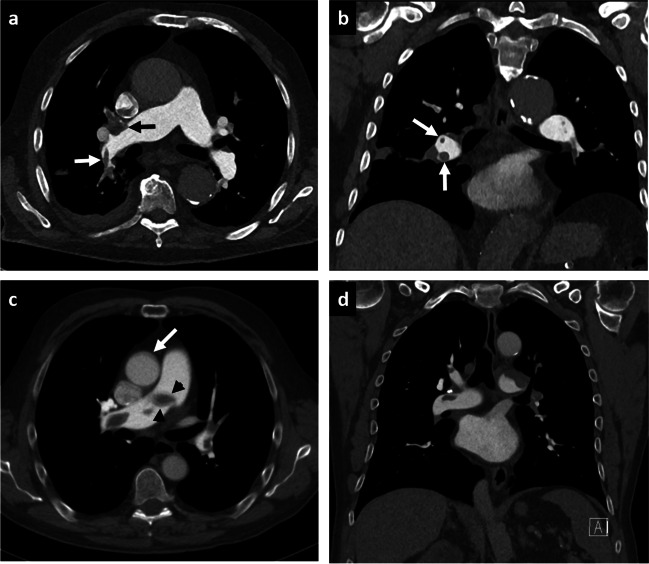
PCD-CT (**a**, **b**) and EID-CT (**c**, **d**) demonstrating bilateral pulmonary embolism. Dual-energy EID-CT exhibits significant pulsation artifacts (**c**; arrow) and blurring of thrombus material (**c**; arrowheads) compared with high-pitch 60-keV PCD-CT. Despite application of a low-dose protocol, PCD-CT delivers excellent intraluminal contrast, paired with high image sharpness and delineation of the thrombus material (**a**, **b**; arrows). *PCD-CT*, photon-counting detector CT; *EID-CT*, energy-integrating detector CT

All CTPAs in both groups were of diagnostic image quality. For 60 keV images, the CTPA image quality was considered excellent or good in 31/32/32/30 cases (96.9%/100%/100%/93.8%; R1/R2/R3/R4) in the PCD group and in 31/30/32/30 cases (96.9%/93.8%/100%/93.8%) in the EID group. An image example is given in Fig. 1, demonstrating CTPA with bilateral pulmonary embolism with PCD and EID at 60 keV. For T3D/blended images, the CTPA image quality was rated as excellent or good in 30/32/28/29 cases (93.8%/100%/87.5%/90.6%) in the PCD group and in 31/32/32/30 cases (96.9%/100%/100%/93.8%) in the EID group. For each group, the overall percentage of CTPAs rated with a subjective image quality score of “1” or “2,” indicating excellent or good image quality, was calculated with the following results: in the PCD group, 93.8% of 60 keV CTPAs and 90.6% of T3D CTPAs were rated with a subjective image quality score of “1” or “2,” while in the EID group 84.4% of 60 keV CTPAs and 90.6% of blended (120 kV) CTPAs were rated with a subjective image quality score of “1” or “2.” Subjective evaluation of image quality is summarized in Table 4.

**Table 4:** Subjective image qu﻿ality

	PCD-CT				EID-CT			
	T3D				Blended (120 kV)			
	60 keV				60 keV			
Likert scale	Reader 1	Reader 2	Reader 3	Reader 4	Reader 1	Reader 2	Reader 3	Reader 4
1	16 (50%)	16 (50%)	18 (56.3%)	12 (37.5%)	18 (56.3%)	28 (87.5%)	23 (71.9%)	15 (46.9%)
2	14 (43.8%)	16 (50%)	10 (31.3%)	17 (53.1%)	13 (40.6%)	4 (12.5%)	9 (28.1%)	15 (46.9%)
3	2 (6.3%)	0 (0%)	4 (12.5%)	3 (9.4%)	1 (3.1%)	0 (0%)	0 (0%)	2 (6.3%)
4	0 (0%)	0 (0%)	0 (0%)	0 (0%)	0 (0%)	0 (0%)	0 (0%)	0 (0%)
ICC	0.71				0.67			
	60 keV				60 keV			
Likert scale	Reader 1	Reader 2	Reader 3	Reader 4	Reader 1	Reader 2	Reader 3	Reader 4
1	21 (65.6%)	21 (65.6%)	27 (84.4%)	19 (59.4%)	11 (34.4%)	20 (62.5%)	23 (71.9%)	18 (56.3%)
2	10 (31.3%)	11 (34.4%)	5 (15.6%)	11 (34.4%)	20 (62.5%)	10 (31.3%)	9 (28.1%)	12 (37.5%)
3	1 (3.1%)	0 (0%)	0 (0%)	2 (6.3%)	1 (3.1%)	2 (6.3%)	0 (0%)	2 (6.3%)
4	0 (0%)	0 (0%)	0 (0%)	0 (0%)	0 (0%)	0 (0%)	0 (0%)	0 (0%)
ICC	0.72				0.47			

*PCD-CT* photon-counting detector CT, *EID-CT* energy-integrating detector CT, *ICC* intraclass correlation coefficient

Rating scores: 1 = excellent image quality, 2 = good image quality, 3 = moderate to poor image quality, 4 = non-diagnostic

According to Koo et al, the intraclass correlation coefficient suggested moderate reliability for ratings of PCD T3D (ICC 0.71), for ratings of PCD scans at 60 keV (ICC 0.72), and for ratings of blended EID scans at 120 kV (ICC 0.67). In contrast, ratings of EID VMI at 60 keV demonstrated poor reliability with an ICC of 0.47 [21].

### Radiation dose

The mean ED (1.4 ± 0.5 mSv [PCD] *vs*. 3.3 ± 0.8 mSv [EID]), as well as SSDE (3.1 ± 0.8 mGy [PCD] *vs.* 5.7 ± 2.2 mGy [EID]) and DLP (80.0 ± 25.3 mGy·cm [PCD] *vs*. 181.7 ± 101.8 mGy·cm [EID]) were significantly lower in the PCD group (all *p* < 0.001). Detailed results of all evaluated radiation dose parameters are summarized in Table 5.

**Table 5:** Radiation dose parameters

	PCD-CT	EID-CT	*p* value
DLP (± SD) [mGy·cm]	80.0 (± 25.3)	181.7 (± 101.8)	< 0.001
ED (± SD) [mSv]	1.4 (± 0.5)	3.3 (± 1.8)	< 0.001
SSDE (± SD) [mGy]	3.1 (± 0.8)	5.7 (± 2.2)	< 0.001

*PCD-CT* photon-counting detector CT, *EID-CT* energy-integrating detector CT, *DLP* dose length product, *ED* effective dose, *SSDE* size-specific dose estimate

## Discussion

In this study, we assessed objective and subjective image quality criteria for an ultra-low contrast medium and radiation dose CT pulmonary angiography scan protocol performed on a first-generation clinical photon-counting detector CT. Our results indicate that despite drastic reductions in both contrast medium and radiation dose administered, the resulting image quality is suitable for the diagnostic workup of patients with suspected acute pulmonary embolism.

**Fig. 2:**
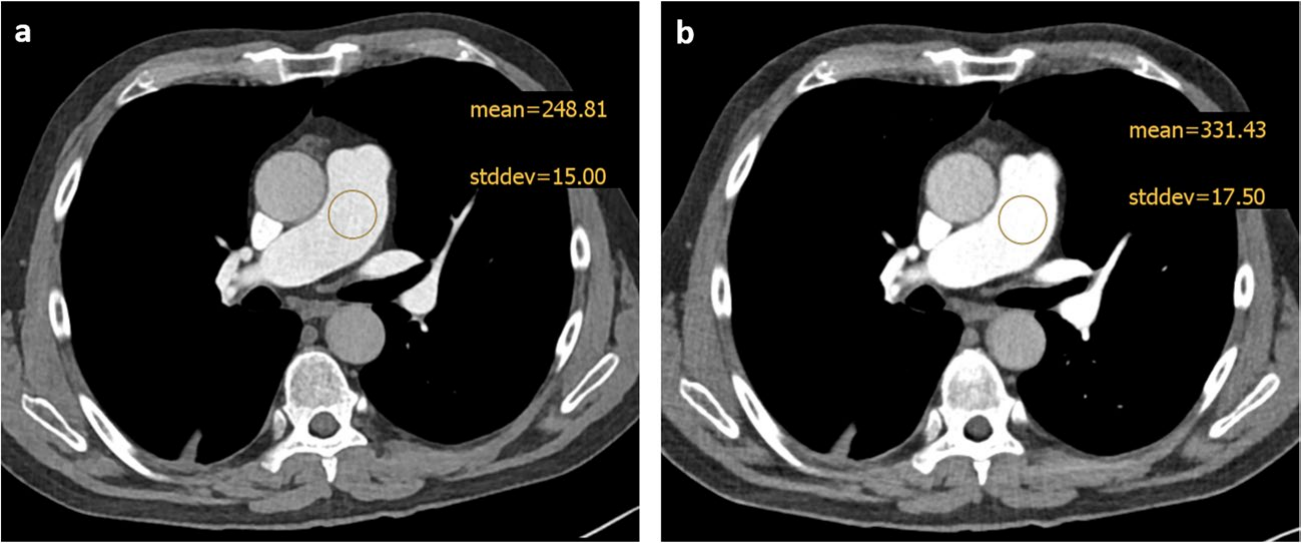
PCD-CT “T3D” (**a**) *vs*. 60-keV images (**b**): compared to polychromatic low-energy threshold 120 kV images—“T3D” (**a**), 60-keV images (**b**) provide significantly higher iodine contrast (mean) at similar noise (stddev) levels. *PCD-CT*, photon-counting detector CT

According to the ALARA (“as low as reasonably achievable”) principle, there is strong clinical interest to keep both contrast agent dose and radiation dose as low as possible. PCD technology offers considerable advantages over standard EID scanners in this respect. First clinical experiences with PCD in cardiovascular imaging have recently been published investigating CT angiography scans of the aorta and the coronary vessels [22–24]. In addition, several studies have been conducted concerning (ultra)-low contrast medium CTPA protocols using conventional EID technology. For example, Rajiah et al evaluated a 30-mL protocol on a 128-slice dual-source EID scanner with promising results [25]. Brendlin et al suggested a contrast-enhanced ultra-low radiation dose high-pitch CT protocol with reduced scan range resulting in an effective radiation dose of 0.7 ± 0.3 mSv [25, 26], while Lu et al evaluated a high-pitch CTPA with iterative reconstruction at 80 kVp and as little as 20 mL of contrast medium on a dual-source EID-CT scanner [18]. Notably, a disadvantage of conventional EID-based single-energy examinations is the inability to perform monoenergetic extrapolarization to enhance the iodine contrast in case of suboptimal vascular opacification. Moreover, spectral image information for the calculation of iodine distribution maps, which show incremental advantages in the diagnosis of pulmonary artery embolism, is lacking [5]. The EID-based DE technology has experienced a considerable hype in the past 10 years, mostly due to decisive advantages over conventional single-energy acquisition [5, 6, 10, 27]. However, whether this acquisition technology is associated with an increased radiation dose remains in question [28–30]. Other restrictions, such as a limited spectral FOV in conventional detector dual-source approaches [11], which can be critical in obese patients, must also be considered (Table 2). In contrast to conventional EID scanners, PCD scanners offer the benefit to combine all the requirements for an ideal CT examination: Full FOV spectral data acquisition (50 cm), low contrast medium, and low radiation dose, as well as the full spectrum of spectral image post-processing applications (Fig. 2).

**Fig. 3:**
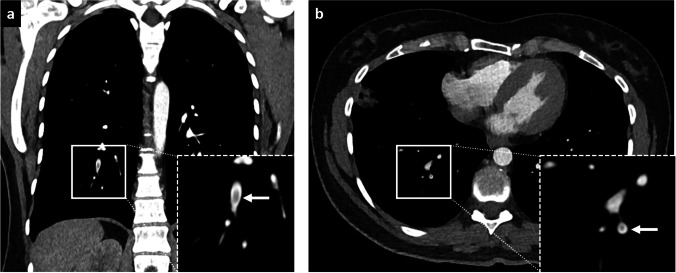
Low-dose PCD-CT demonstrating peripheral pulmonary embolism at segment level in the right lower lobe at coronal (**a**) and corresponding axial (**b**) 60-keV reconstructions. The minute contrast medium filling defect is magnified and highlighted by arrows. *PCD-CT*, *photon-counting detector CT*

To the authors’ best knowledge, this is the first study investigating a PCD for CTPA. We were able to show that the acquisition of CTPAs on a PCD-CT scanner with a total of 25 mL of iodinated contrast medium is feasible while maintaining excellent to good image quality in nearly 100% of examinations. The option of significantly reducing the volume of contrast medium (in our case by half compared to our in-house standard EID protocol) is especially advantageous in patients with kidney preconditions, since the risk of contrast-medium induced nephropathy increases with the applied volume of contrast medium [31]. A lowered iodine delivery rate can be compensated in parts by the increased image contrast due to virtual low-kV imaging [32–34]. Furthermore, our results indicate that a reduction of effective radiation dose by 51.2% (1.4 mSv *vs*. 3.3 mSv in the EID group) can be realized without a loss of subjective image quality or diagnostic confidence (Fig. 3). Notably, assessed objective image quality parameters were superior in the EID group. According to our experience, the decrease in contrast-to-noise-ratio between detector systems is acceptable in any case, especially when taking higher resolution, less motion artifacts, and the achieved severe dose savings achieved with the PCD system into account. With a mean of 80.0 mGy·cm, DLP in the PCD group was significantly lower in comparison to the usual radiation exposure for CTPA in Europe (138 mGy·cm) and the USA (420 mGy·cm), as recently evaluated by Bos et al [35].

Subjective image quality was considered best by all readers for 60 keV PCD examinations with excellent or good ratings in 93.8% of PCD-CTPAs. The main reason for higher subjective image ratings in the PCD group was lower susceptibility to motion and especially breathing artifacts. In other words, images derived from conventional EID-based dual-energy CT often appear blurry (Fig. 1). Technically, this is mainly due to the fact that the helical pitch factor for dual-energy acquisitions on conventional EID scanners is limited by the vendors (in case of the EID system used in this study default setting of 0.55), whereas the PCD-equipped scanner allows for multi-energy data acquisition with higher pitch factors (2.0 in our study setting) associated with significantly shorter scan times. This issue is especially advantageous for patients who have difficulty following breath-holding commands due to dyspnea, which would be a quite expected symptom in patients presenting with pulmonary embolism [1]. No CTPA examination was deemed “non-diagnostic” on either scanner, irrespective of the acquisition or reconstruction settings.

Our study has several limitations: First, we did not evaluate the diagnostic accuracy of the ultra-low contrast medium PCD-CTPA protocol. However, the similar frequency of detected pulmonary embolisms (10 *vs.* 7 out of 32 CTPAs in the PCD/EID group) and diagnostic image quality ratings in all examinations suggest that PCD-CTPA most likely is not inferior to the EID group. Second, the iodine delivery rates differed between study groups. Moreover, the reconstruction kernels between detector systems differed slightly. Third, with less pulsation artifacts and the shorter contrast medium bolus in the PCD group, image impression of PCD-CTPAs varied from EID-CTPAs; thus, readers may have become accustomed to either scanners’ characteristics despite being blinded for their subjective image quality reading. Last, at the time of image acquisition and data evaluation, no dedicated software was available for calculating iodine distribution maps from PCD-CTPA data. Therefore, image quality and diagnostic value of suchlike maps could not be evaluated for PCD-CTPA in the current study, warranting further investigations in that regard.

## Conclusion

High-pitch photon-counting CT pulmonary angiography allows for significant reduction of contrast medium and radiation dose in the diagnosis of acute pulmonary embolism, while maintaining good to excellent image quality in comparison with dual-energy pulmonary angiography on a conventional CT scanner with energy-integrating detector technology.

### Supplementary Information

Below is the link to the electronic supplementary material.

Supplementary file1 (PDF 145 KB)

## Abbreviations

CNR: Contrast-to-noise ratio
CTDI: Computed tomography dose index
CTPA: Computed tomography pulmonary angiography
DE: Dual-energy
DLP: Dose-length product
ED: Effective dose
EID: Energy-integrating detector
FOV: Field of view
ICC: Intraclass correlation coefficient
PCD: Photon-counting detector
PE: Pulmonary embolism
ROI: Region of interest
SNR: Signal-to-noise ratio
SSDE: Size-specific dose estimates
VMI: Virtual monoenergetic imaging

## Declarations

### Guarantor

The scientific guarantor of this publication is Bernhard Petritsch.

### Statistics and biometry

One of the authors (Andreas M. Weng) has significant statistical expertise.

### Study subjects or cohorts overlap

None of the study subjects or cohorts have been previously reported.

### Methodology

• Retrospective

• Diagnostic study

• Performed at one institution
