# Supplementary material for: Photon-counting CT for diagnosis of acute pulmonary embolism: potential for contrast medium and radiation dose reduction
Source: Eur Radiol. 2023 Jun 14;33(11):7830–9. doi: 10.1007/s00330-023-09777-9 (PMC10598187; doi:10.1007/s00330-023-09777-9)

Supplementary Figure

60 keV PCD-CT images acquired with different (in terms of decreasing) CM dose. Primarily patients were acquired with 50ml CM (**a**; matching CM protocol to EID). CM dose was first lowered to 35ml (**b**) and to 30ml (**c**) in a second step. Finally 25 ml (**d**) proved to be sufficient to achieve adequate vessel opacification in most different patients. *PCD-CT = photon-counting detector CT; CM = contrast medium*

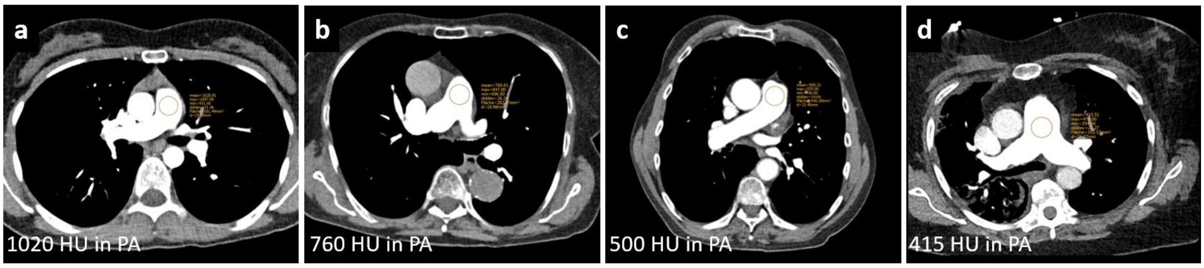

Supplement: Supplementary file 1 — Supplementary file1 (PDF 145 KB) [file 330_2023_9777_MOESM1_ESM.pdf]
